# Smart composite nanofiber mats with thermal management functionality

**DOI:** 10.1038/s41598-021-83799-5

**Published:** 2021-02-19

**Authors:** Nuray Kizildag

**Affiliations:** Integrated Manufacturing Technologies Research and Application Center, Sabanci University, Tuzla, 34956 Istanbul, Turkey

**Keywords:** Synthesis and processing, Synthesis and processing

## Abstract

Nanofibers with thermal management ability are attracting great attention in both academia and industry due to the increasing interest in energy storage applications, thermal insulation, and thermal comfort. While electrospinning is basically a fiber formation technique, which uses electrostatic forces to draw ultrafine fibers from a wide variety of polymers, with the addition of phase change materials (PCMs) to the electrospinning solution it enables the production of shape stabilized phase change materials with thermal management functionality. In this study, polyacrylonitrile (PAN) nanofibers containing paraffinic PCMs were produced by electrospinning method and the composite nanofibers obtained were characterized in terms of their morphology, chemical structure, thermal properties, stability, thermal degradation behaviour and hydrophobicity. Besides, PCMs with different phase transition temperatures were added simultaneously into the nanofiber structure in order to investigate the tunability of the thermoregulation properties of the nanofibers. Uniform nanofibers with thermal management functionality were obtained. It could be possible to obtain composite nanofibers showing thermoregulation ability over a wider temperature range by simultaneous addition of PCMs with different melting points into the nanofiber structure. 50 wt% PCM could be added to PAN nanofiber structure wherein the resulting nanofiber exhibited 58.74 J g^−1^ of enthalpy storage during heating and 57.41 J g^−1^ of heat release during cooling. The composite nanofibers maintained their cylindrical fiber morphology, structure and composition after multiple heating–cooling cycles and retained their thermal management functionality. The contact angle measurements showed that the addition of PCMs imparted hydrophobicity to the nanofibers.

## Introduction

Being one of the basic elements of clothing comfort, the thermo-physiological aspect includes the heat and moisture transmission characteristics of clothing. Thermo-physiologically comfortable clothing supports the thermoregulation of the body and helps the wearer to keep a comfortable temperature. In this regards, new textile concepts have been introduced, one of which is the addition of PCMs to the structure of textile materials^1,2^. PCMs are materials, which change phase at certain temperatures and accordingly have the ability to absorb, store and release large amounts of energy in the form of latent heat depending on the surrounding temperature^1–5^. PCMs can be grouped into three major classes such as organic compounds, inorganic compounds, and eutectic compounds of the first two. While organic PCMs are polyethylene glycols (PEGs), paraffin waxes, and fatty acids, inorganic PCM compounds consist of metals, metal alloys, salts, and salt hydrates. Melting temperature, temperature range of the phase change and heat storage capacity are important factors for a PCM and the selection depends on the end application^5^.

When added to textile materials, PCMs improve the thermal comfort of textile materials by providing proactive temperature management which is especially preferred in some applications such as everyday clothing (sportswear, diving suit, skiwear, underwear), professional clothing (firefighters’ suit, space suits, sailor suits), interior decoration and essentials (curtains, beddings, pillows, mattresses, sleeping bags), shoe linings, automotive textiles (seat covers) and is of vital importance in some other applications such as surgical gloves, bedding materials, bandages and products which are used in hospitals and especially in intensive care units to regulate patients’ temperatures and keep within the comfort range^3,6^. Although majority of the regulation is done by skin, textile materials with thermoregulation properties can be used to help the skin preserve its temperature within the comfort zone, especially during abnormal fluctuations of temperature^1^. Thermoregulation properties can be achieved in textile materials by different ways: Textile fibers can be functionalized by mixing/blending PCMs into the fiber spinning solution/melt^7^, loading PCMs into the core of hollow fibers^8,9^ or incorporation of PCMs to the polymer chain during synthesis^10^. PCMs can also be applied to fabrics by coating^11,12^ or finishing processes^13–15^. Electrospinning is another preferred technique especially in research studies showing the feasibility of producing textile materials with thermal management properties^16–25^.

Electrospinning is a simple and cost-effective technique to produce fibers from a wide variety of polymers with diameters ranging from nanometres to submicrons. It is a unique approach that uses electrostatic forces to produce fibers from polymer solutions (or melts). The polymer solution filled into a syringe is introduced to the tip of a needle to form a pendant drop by a syringe pump, charged by the application of high voltage and then subjected to electric field. When the electric field overcomes the surface tension of the polymer solution, a charged jet ejects from the tip of needle, undergoes unstable whipping and bending motions between the tip of the needle and the collector, where the solvent evaporates and polymer in the nanofiber form is collected on the collector^26–28^. Electrospun nanofibers have attracted great attention due to their ease of fabrication, unique properties such as high surface-area-to-volume ratio, low density, and high pore volume and possibilities of functionalization^29–31^ and used in a wide range of applications such as filtration^32^, energy harvesting and storage^33^, tissue engineering^34^, drug delivery^35^, wound healing^36^, sensors^37^, and polymer reinforcement^38^, etc. Composite nanofibers produced with the addition of different types of additives displayed additional functionalities. It has been possible to produce antistatic nanofibers with polyaniline^39^, antibacterial nanofibers containing silver nanoparticles^40^, and antistatic nanofibers showing enhanced tensile properties with the addition of carbon nanotubes^41^.

It has also been possible to produce composite nanofibers with thermoregulating properties by adding PCMs into the nanofiber structure. The studies on the production of composite nanofibers containing PCMs mainly focused on the use of individual fatty acids, their eutectics, and polyethylene glycols^16–25^. Chen et al. conducted a series of studies on the incorporation of fatty-acids and fatty-acid derivatives as PCMs into nanofiber structure using conventional electrospinning^16–18^. They successfully electrospun nanofibers of polyethylene terephthalate (PET)/lauric acid, which had smooth surfaces and sufficient tensile strength using conventional electrospinning. With their highest melting enthalpy of about 70.76 J g^−1^, the nanofibers were suggested for applications such as thermo-regulating textiles and solar energy storage^16^. They also produced composite PET nanofibers using different types of fatty acids such as lauric acid, myristic acid, palmitic acid, and stearic acid as additives. The morphology and the average diameter of the nanofibers were affected by the amount of the fatty acid. The thermal storage ability and the thermal transition temperatures observed were dependent on the type of the fatty acids. While the melting temperature obtained varied between 45 and 69 °C, heat of fusion varied between 54.91 and 67.88 J g^−1^^18^. Producing composite nanofibers of polyamide/lauric acid, Cai et al. reported about the formation of ribbon shaped nanofibers with melting enthalpies changing between 63.66 and 74.12 J g^−1^ as the fatty acid content increased from 80 to 150%^19^. Producing PET nanofibers with a series of different fatty acid eutectics in another study, they reported that the latent heat and phase transition temperature of composite fibers were dependent on the type of the fatty acids. Thermal analysis suggested that the melting and crystallization enthalpies of composite fibers increased gradually with increasing fatty acid eutectic amounts, with melting enthalpies ranging from 33.91 to 112.7 J g^−1^^20^. Ke et al. electrospun composite PET nanofibers using a series of fatty acid eutectics for thermal energy storage. PET/PCM ratios were selected as 100/50, 100/100, and 100/150. The composite nanofibers’ diameter ranged from 100 to 300 nm and they showed different thermal behaviours depending on the type and content of PCMs. The melting enthalpies observed were between 34.18 and 95.24 J g^−1^^21^. Using PEG as additive, Chen et al. produced cellulose acetate/PEG nanofibers. According to thermal analysis, the heat capacities of the composite fibers with 10–70 wt% PEG varied between 3.8 and 120.2 J g^−1^^22^. Additionally, they studied the effects of molecular weight of PEG on morphology, thermal properties, and mechanical properties of the composite fibers. They used PEG with five different molecular weights, which varied between 2000 and 20,000 g mol^−1^. According to the thermal analysis results, the composite fibers displayed thermal storage and release properties in different temperature ranges, which were dependent on the molecular weight of PEG. The melting enthalpy of the composite fibers varied between 53.23 and 86.03 J g^−1^ depending on the molecular weight of PEG^23^. Hu et al. produced core–shell nanofibers using natural soy wax (bio-based PCM) as the core and polyurethane as the shell by coaxial electrospinning. Uniform fiber morphology with a core–shell structure, and a homogeneous wax distribution throughout the core of the fibers, were obtained. Thermal analysis results showed that the enthalpy increased with wax content. The melting enthalpy increased from 9.24 to 36.47 J g^−1^ as the wax content in the fiber structure increased from 10 to 60%^24^.

Although there are many studies in literature investigating the properties of PCMs, composite PCM materials and composite nanofibers with fatty acids and polyethylene glycols, there is very limited number of studies showing the use of pure paraffins in the production of functional nanofibers with thermal management functionality. In this study, two different PCMs, both of which were 100% paraffin with different thermal transition temperatures, were directly added into PAN nanofiber mats to impart thermal regulation properties to nanofibers. They were also added simultaneously to the nanofiber structure to investigate the possibility of tuning the thermoregulation properties (impart higher heat storage/release capacity over a wider temperature range). The composite nanofibers were analysed comprehensively regarding their morphology, chemical structure, thermal properties, stability, thermal degradation behaviour and hydrophobicity. This study provides substantial contribution to the literature by showing the development of chemically, morphologically and thermally stable composite PAN nanofibers with thermoregulation properties, pointing out the required process parameters for the production of uniform-structured PAN/PCM nanofibers, showing the possibility of finetuning the properties of these nanofibers by incorporating different types of PCMs simultaneously into the nanofiber structure and changing their ratios in order to obtain higher storage/release capability over a wider temperature range, and providing comprehensive discussion regarding morphology, chemical structure, thermal properties, stability, thermal degradation behaviour and hydrophobicity of the composite PAN nanofibers containing PCMs.

## Experimental details

### Materials

PAN (181315, average Mw: 150.000 g mol^−1^), provided by Sigma Aldrich, was used as the fiber forming polymer. Dimethylformamide (DMF) was supplied from Sigma Aldrich and used as the solvent. PCMs with two different melting points of 28 °C (PCM28) and 32 °C (PCM32) were provided by Microtek Laboratories, Inc. and added to the nanofiber structure in order to obtain nanofibers with thermal management functionality. The properties of the PCMs provided by the manufacturer are presented in Table 1.

**Table 1:** Properties of PCMs used in the production of composite PAN/PCM nanofiber mats.

Properties	PCM28	PCM32
Appearance	Liquid and colourless about melting point; solid and opaque below freeze point	Liquid and colourless about melting point; solid and opaque below freeze point
Form	Bulk	Bulk
Type	Paraffin	Paraffin
PCM content	100%	100%
Melting point	28 °C	32 °C

### Methods

#### Preparation of the electrospinning solutions

The required amounts of PCMs were added to the required amount of N,N-dimethylformamide (DMF). Ultrasonic tip was utilized for the homogenization of PCM/DMF dispersions. After homogenization of the dispersions with ultrasonic tip for 10 min, PAN was added to the dispersion and dissolved by stirring magnetically for 3 h at 40 ℃. The concentration of PAN was 10 wt%. Reference nanofibers were also produced from 10 wt% PAN/DMF solution.

#### Electrospinning

**Figure 1:**
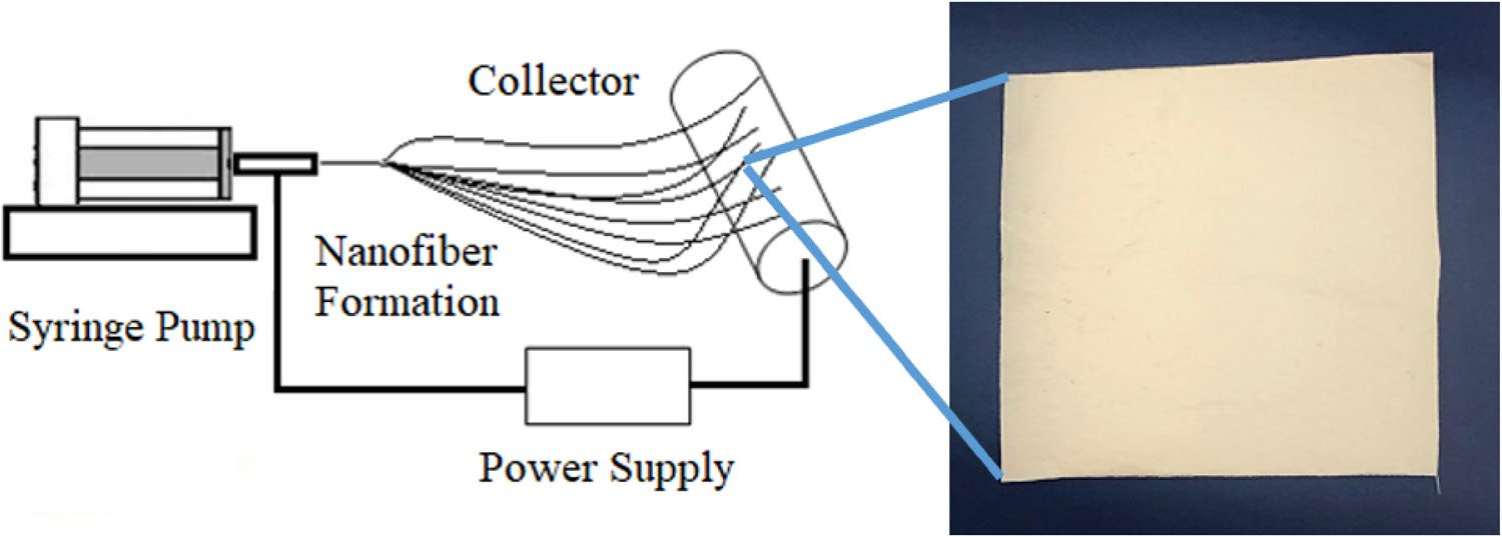
Schematic of the electrospinning setup used in the production of the composite PAN/PCM nanofibers and photograph of composite PAN nanofibers with 50% PCMs (PAN-50PCM28-50PCM32).

Composite nanofibers containing PCMs were produced on horizontal electrospinning setup. Illustrated in Fig. 1, the electrospinning setup, which was used in this study, contained a syringe pump (SINO MDT, SN-50C6), a high voltage power supply (Matsusada, 0–50 kV), and a grounded rotating collector.

Composite electrospinning solutions of PAN/DMF with PCMs were fed through a blunt needle with a diameter of 1.25 mm, using a syringe of 10 mL. The nanofibers were collected on a rotating metal collector which was covered by nonwoven, respectively. The distance between the tip of the needle and the collector was set as 10 cm, the flow rate as 1 mL h^−1^ and the applied voltage as 15 kV. Electrospinning was performed under standard atmospheric conditions (Temperature: (20 ± 2) ℃, Relative humidity: (65 ± 5) %). The nanofiber mats were collected for 4 h. The compositions of nanofiber mats produced are presented in Table 2.

**Table 2:** Compositions of the PAN/PCM nanofiber mats produced.

Samples	wt% of PCM28 (based on PAN weight)	wt% of PCM32 (based on PAN weight)	Total PCM content (wt%) (based on composite web weight)
Neat PAN	–	–	–
PAN-25PCM28	25	–	20
PAN-50PCM28	50	–	33
PAN-25PCM32	–	25	20
PAN-50PCM32	–	50	33
PAN-12.5PCM28-12.5PCM32	12.5	12.5	20
PAN-25PCM28-25PCM32	25	25	33
PAN-50PCM28-50PCM32	50	50	50

#### Characterization

The viscosity and conductivity of PAN/DMF and PAN/%50PCM/DMF electrospinning solutions were measured. The viscosity was measured with Brookfield DV2TR using SC4-21 spindle at 50 RPM. The conductivity was measured using Hanna HI 8633 conductivity meter. Scanning electron microscope (SEM) (Jeol Quanta 200 FESEM) was used to analyse the morphology of the nanofibers. Samples were coated with gold using a sputter coater before SEM analysis. SEM images were taken at an accelerating voltage of 20 kV. The fiber diameters were measured from the SEM images using Image J. The average nanofiber diameters and the standard deviations were based on 50 measurements per sample. Average nanofiber diameters were expressed as the mean ± standard deviation (S.D.). In addition, SEM was also utilized to show the form stability of composite nanofibers after 50 consecutive heating–cooling cycles. Fourier Transform Infrared Spectrometer (FTIR) (Perkin Elmer-Spectrum Two) was used to record absorption spectra of composite nanofibers in a range from 4000 to 650 cm^−1^ with a resolution of 4 cm^−1^. 32 scans were taken for each experiment and averaged to obtain the FTIR spectra of composite PAN/PCM nanofiber mats. FTIR was also utilized to investigate the chemical structure of composite nanofiber mat after 50 consecutive thermal cycles. Differential Scanning Calorimetry (DSC) (Mettler Toledo) was used to investigate the thermal properties of PCMs, neat PAN and composite PAN/PCM nanofiber mats. PCMs were tested in a temperature range from 0 to 50 °C, with a heating/cooling rate of 5 ºC min^−1^, under nitrogen (N_2_) with a flow rate of 50 ml min^−1^ in order to understand their phase transition properties such as thermal transition temperatures and enthalpies. The composite nanofibers were also tested in a temperature range from 0 to 50 ºC, with a heating/cooling rate of 5 ºC min^−1^ in nitrogen environment in order to evaluate the latent heat storage and release ability of the nanofibers. The thermal characteristics were evaluated from the second heating of the samples. Besides, 50 consecutive heating–cooling cycles between 0 and 50 °C with a heating rate of 5 °C min^−1^ were applied to both PCMs and composite PAN nanofiber mat with 50% PCM32 monitor the variations in the latent heat storage and release ability of the nanofibers by repeated thermal cycles and evaluate the thermal stability of the composite nanofibers. The phase transition temperature was taken as the peak point of the DSC curves. The enthalpy of melting (ΔH_m_) and the enthalpy of crystallization (ΔH_c_) were calculated based upon the areas under the solid–liquid and liquid–solid phase change peaks of paraffins using the thermal analysis software affiliated with the equipment. Thermogravimetric analysis (TGA) (Mettler Toledo) was used to determine the thermal decomposition behaviours of PCMs, neat PAN and composite PAN/PCM nanofiber mats. The heating rate was set as 10 °C min^−1^ and the TGA curves were recorded from room temperature to 1000 °C under N_2_ flow with a rate of 50 ml/min. Derivative TGA (DTGA) curves were obtained from the TGA curves. Water contact angle measurements were also conducted to characterize the nanofiber mats in terms of hydrophobicity.

## Results and discussion

The results obtained are discussed below with the help of the relevant tables and figures.

### Morphology of neat PAN and composite PAN/PCM nanofiber mats

**Figure 2:**
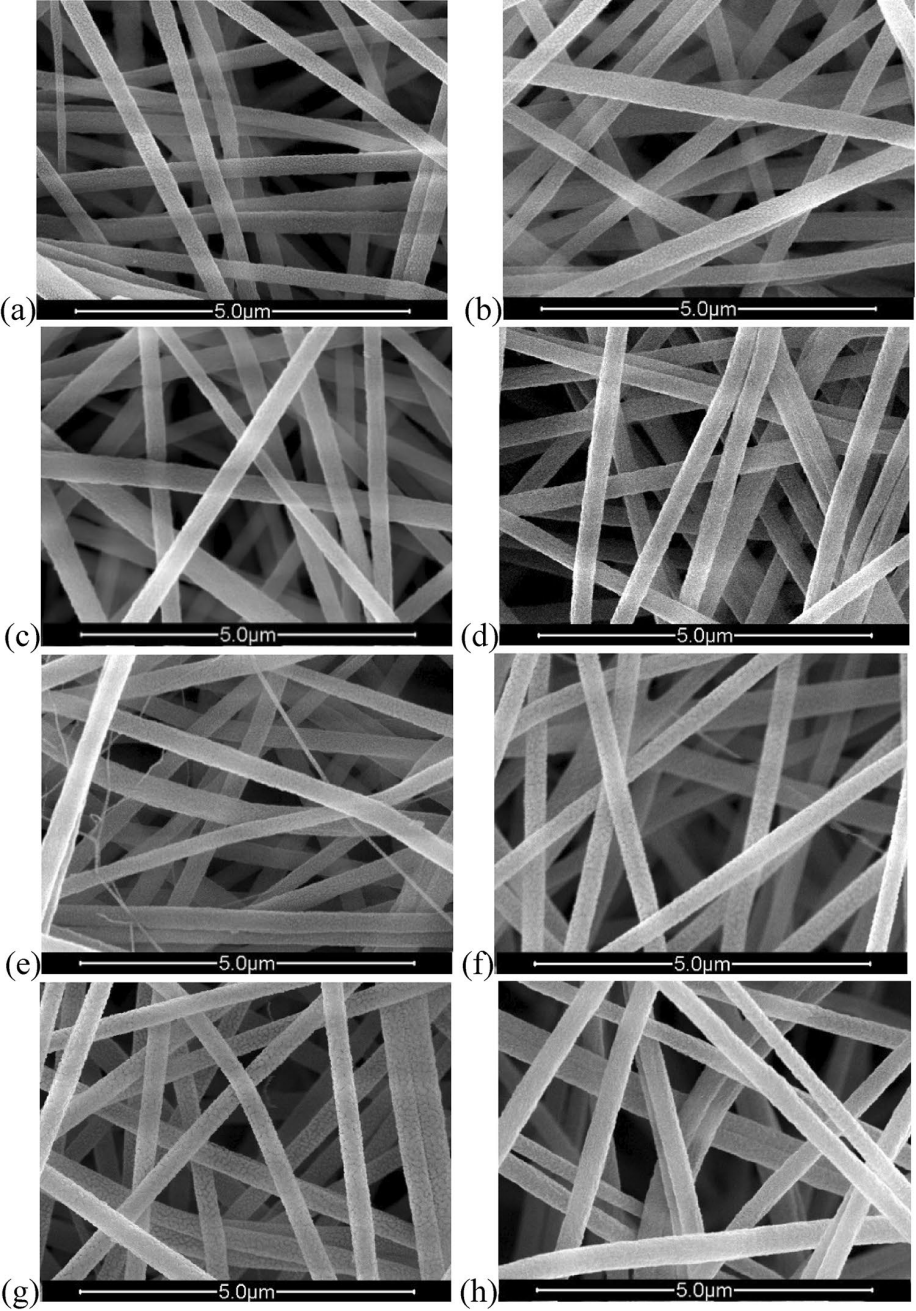
SEM images of (**a**) neat PAN; (**b**) PAN-25PCM28; (**c**) PAN-50PCM28; (**d**) PAN-25PCM32; (**e**) PAN-50PCM32; (**f**) PAN-12.5PCM28-12.5PCM32; (**g**) PAN-25PCM28-25PCM32; (**h**) PAN-50PCM28-50PCM32.

SEM images of neat PAN and composite PAN/PCM nanofiber mats, taken with 25 kX magnification, are presented in Fig. 2 while the average nanofiber diameters are given in Table 3.

**Table 3:** Average diameters of neat PAN and composite PAN/PCM nanofibers.

Samples	Average diameter (nm) ± S.D.
Neat PAN	257.15 ± 39.18
PAN-25PCM28	301.39 ± 42.67
PAN-50PCM28	324.47 ± 58.54
PAN-25PCM32	284.45 ± 29.86
PAN-50PCM32	325.60 ± 38.30
PAN-12.5PCM28-12.5PCM32	283.32 ± 52.50
PAN-25PCM28-25PCM32	319.28 ± 38.32
PAN-50PCM28-50PCM32	350.15 ± 28.40

The white colour of the nanofiber mats (Fig. 1) and also the fiber forms were maintained after PCM addition. The SEM observations from the electrospun PAN/PCM composite nanofiber mats revealed that the nanofibers were cylindrical in shape and had a smooth external surface, free of any beads. The nanofibers were uniform in structure even at the 50 wt% PCM addition (PAN-50PCM28-50PCM32).

SEM images showed that the diameters of the nanofibers were affected by the incorporation of additives. From the previous studies, morphology of the electrospun fibers are primarily influenced by the type of polymer, the solution properties and the process parameters^17^. Since polymer concentration and the electrospinning parameters were fixed in this study, the morphology of the electrospun fibers considered to be affected mainly by the PCM content. The effects of the incorporation of additives into nanofiber structure are widely discussed in literature. Jo et al. reported an increase in fiber diameter with titanium dioxide addition^42^. In many studies, decrease in nanofiber diameter was reported with silver nitrate addition^43–45^. The addition of fatty acids and PEG and increase in their contents were reported to result in larger nanofiber diameters^16,18–20,46^.

The viscosity and the conductivity of the electrospinning solutions are two important parameters that have effects on nanofiber diameter and are affected by addition of additives to a great extent. While increase in viscosity of the electrospinning solution leads to the formation of thicker nanofibers^47^, increase in conductivity leads to the formation of thinner nanofibers to some extent and then coarser nanofibers after a critical value^31,44,45^. Depending on the compromise between viscosity and conductivity, the average nanofiber diameter is determined. The viscosity and conductivity of PAN/DMF solution were measured as 174 mPa s and 158 µS/cm, respectively. When 50% PCM was added into the PAN solution, the viscosity increased to 332 mPa s and the conductivity remained almost constant (was measured as 150 µS/cm). Thus, the viscosity effect was dominant when PCM was added and as a result of this, increase was observed in average nanofiber diameter. While the neat PAN nanofibers had an average diameter of 257.15 nm, the average nanofiber diameter increased to about 350.15 nm with 50% PCM addition (PCM-50PCM28-50PCM32).

A good shape stabilizer should be able to host a significant amount of PCM to have better thermal management properties since the thermal regulation is performed by those compounds. According to literature, morphology analysis showed that the maximum PCM that could be incorporated into fiber structure changed depending on the PCM type used. While it could be possible to add as much as 100% fatty acids without disturbing fiber structure^16–18^, the maximum with PEG was 70 wt%^22^. In this study, it could be possible to add up to 50% paraffinic PCMs to the nanofiber structure without any deteriorations in the nanofiber and nanofiber mat structure.

### Fourier transform infrared spectroscopy (FTIR)

FTIR spectroscopy was used to confirm the incorporation of PCM into PAN nanofiber structure. Besides, the spectra obtained was used to compare the spectral differences between the composite nanofiber mats and the neat PAN nanofiber mat.

**Figure 3:**
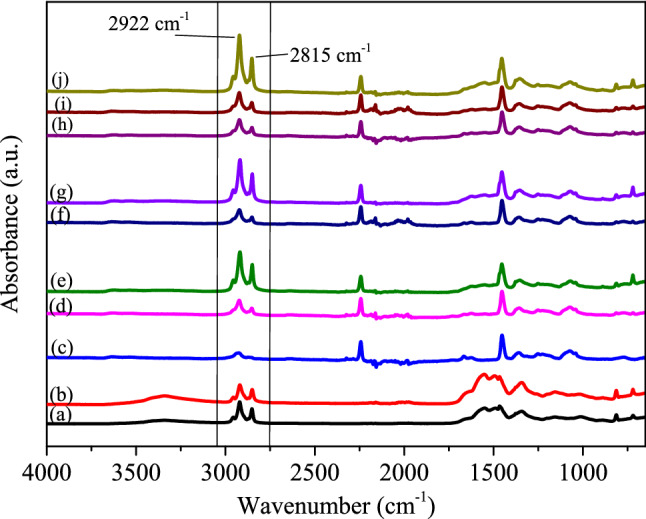
FTIR spectra of (**a**) PCM28; (**b**) PCM32; (**c**) neat PAN; (**d**) PAN-25PCM28; (**e**) PAN-50PCM28; (**f**) PAN-25PCM32; (**g**) PAN-50PCM32; (**h**) PAN-12.5PCM28-12.5PCM32; (**i**) PAN-25PCM28-25PCM32; (**j**) PAN-50PCM28-50PCM32.

The spectra of PCMs were normalized to the largest peak observed, namely the –CH_3_ stretchings observed at 2922 cm^−1^ and the spectra of neat PAN and composite nanofibers were normalized to the characteristic C≡N stretching band of PAN which appears around 2242 cm^−1^ to be able to compare them and obtain comparable absorbance values. The normalized FTIR spectra of PCMs, neat PAN and composite PAN/PCM nanofiber mats are presented in Fig. 3.

**Figure 4:**
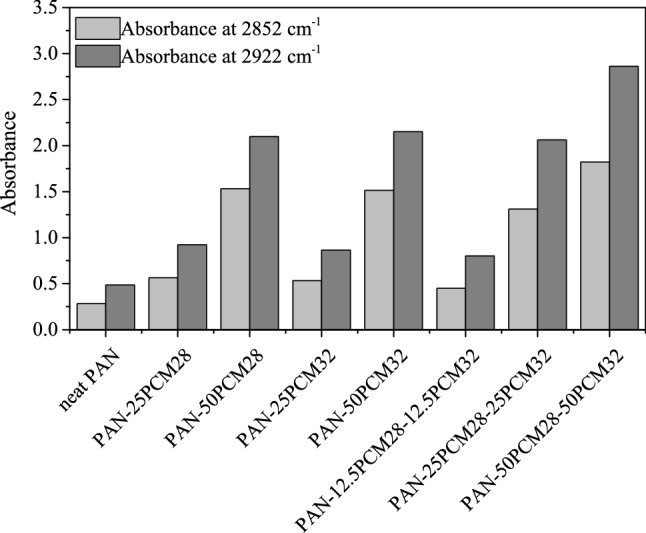
Variations of absorbance values of peaks at 2852 and 2922 cm^−1^.

In Fig. 3a,b, the bands located at 2955, 2922 and 2852 cm^−1^ are the characteristic of the aliphatic C–H stretching vibration. Peaks at 1470 and 1465 cm^−1^ are the bending vibration of the –CH_2_ group; 1375 cm^−1^ is the in-plane bending vibration of C–H and C–C; 728 and 720 cm^−1^ are the rocking vibration of C–H in the paraffin^48,49^. The following peaks are observed in the spectra of PAN nanofibers (Fig. 3c): 3626 cm^−1^ (OH stretching), 2922 and 2870 cm^−1^ (C–H asymmetric and symmetric stretchings in CH, CH_2_, and CH_3_ groups), 2242 cm^−1^ (C≡N stretching), 1622 cm^−1^ (C=C stretching), 1452 cm^−1^ (CH_3_ bending and CH_2_ scissor vibration), 1356 cm^−1^ (CH_3_ symmetric bending vibration in C–CH_3_), and 1252 cm^−1^ (C–N stretching), 1070–1040 cm^−1^ (C = N bending), and 778 cm^−1^ (–C–CN– stretching)^22,45,50,51^. Displaying the characteristic peaks of PCMs in addition to the characteristic peaks of PAN, the FTIR spectra of composite nanofibers, shown in Fig. 3d,e,f,g,h,i,j confirmed the successful incorporation of PCMs into the PAN nanofiber structure. The spectra also showed that there was no chemical reaction between PAN and PCMs. Presented in Fig. 4, the absorbance values of the peaks related with PCMs (located between 2852 and 2922 cm^−1^) increased with the increase in the PCM content.

### Thermal management functionality

For the textile material with thermal management functionality, appropriate phase transition temperatures which are close to skin temperature along with high latent heat storage capability are the most desirable characteristics. In this study PCMs, which had melting points close to the skin temperature were especially chosen. The composite nanofiber mats containing PCMs were analysed with regards to their thermal properties (melting temperature (T_m_), crystallization temperature (T_c_), enthalpy of melting (ΔH_m_), and enthalpy of crystallization (ΔH_c_) of the samples) via DSC and also compared to the PCMs. DSC is an effective method for testing the functionality of PCM materials. It provides information about the amount of energy that can be stored and released by a material. The energy that can be stored is associated with the enthalpy of melting, whereas the energy that can be released is related to the enthalpy of crystallization^52^.

**Figure 5:**
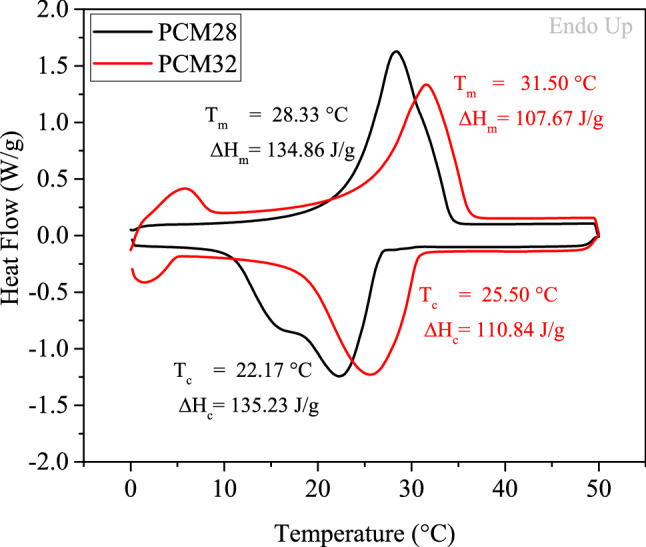
DSC thermograms of PCMs.

DSC thermograms of PCMs are presented in Fig. 5. PCM28 displayed a single endothermic (melting) peak at around 26 °C and double exothermic peaks at around 15 and 23 °C while PCM32 exhibited single endothermic and exothermic peaks at around 29 and 26 °C, respectively. The melting and crystallization enthalpies of PCM28 were measured as 134.86 and 135.23 J g^−1^, respectively. The melting enthalpy of PCM32 was 107.67 J g^−1^ while the crystallization enthalpy was measured as 110.84 J g^−1^. PCM type was expected to be in charge of the intrinsic thermal properties of the novel thermal-storage materials by fixing the phase transition temperatures and limiting the maximum value of the latent heats while the PCM content was expected to play a key role in the latent heats of novel thermal-storage materials.

**Figure 6:**
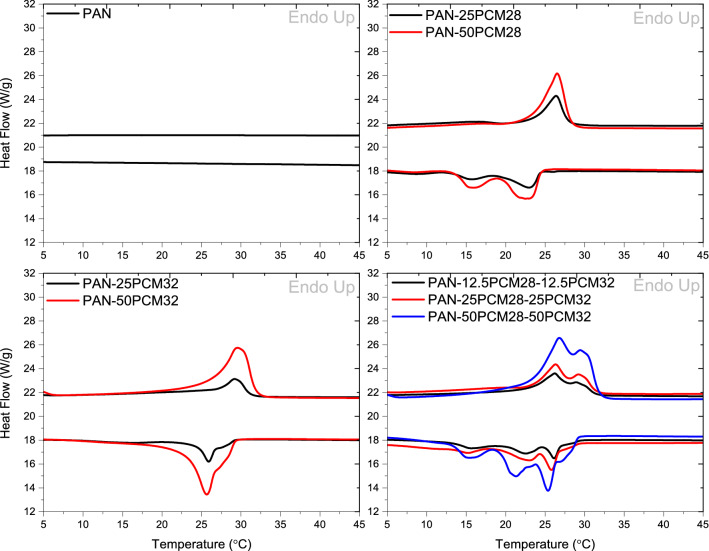
Heating and cooling curves of neat PAN and composite PAN/PCM nanofibers.

DSC thermograms of neat PAN and composite PAN/PCM nanofiber mats are presented in Fig. 6. While no phase transition was observed between 0 and 50 °C for neat PAN nanofibers, composite nanofiber mats showed phase transitions due to the presence of PCMs in the nanofiber structure. The corresponding thermal properties (phase transition temperatures and enthalpy values) of composite nanofiber mats are summarized in Table 4. The phase change temperatures (T_m_ and T_c_) of composite fibers were dominated by the type of PCMs and had no obvious variations compared with those of PCMs. The slight decrease, which was less than 2 ℃, was likely caused by weak molecular interactions between PCMs and PAN nanofiber mat, which affected the required energies for phase transitions to take place and led to the depression of the phase change temperature^53^. On DSC thermograms of the composite nanofibers produced with the simultaneous addition of PCM28 and PCM32, two endothermic peaks and three exothermic peaks resembling the thermal transition behaviour of PCMs were obvious. It could be possible to obtain composite nanofibers showing thermoregulation ability over a wider temperature range by simultaneously adding PCMs with different melting points (PCM28 and PCM32) into PAN nanofiber structure. The composite nanofibers of PAN containing PCM28 and PCM32, displayed thermoregulatory properties between 22.75 and 31.65 °C during heating and between 29.68 and 12.62 °C during cooling which were much wider compared to nanofibers containing only PCM28 and PCM32. Enthalpy storage values and enthalpy release values increased with the increase in the concentration of PCMs. The melting enthalpy increased from 11.42 to 24.71 J g^−1^ and the crystallization enthalpy increased from 11.53 to 26.45 J g^−1^ as the PCM28 content increased from 20 to 33%. For the PCM32 containing composite PAN nanofibers, the melting enthalpy increased from 14.88 to 31.89 J g^−1^ and the crystallization enthalpy increased from 17.16 to 37.64 J g^−1^ as the PCM 32 content increased from 20 to 33%. The highest storing and releasing enthalpy values, which were 58.74 and 57.42 J g^−1^ were observed for composite PAN nanofibers containing the highest amount of PCMs. For the composite nanofibers produced with the simultaneous addition of PCM28 and PCM32, melting enthalpy increased from 13.57 to 58.74 J g^−1^ and the crystallization enthalpy increased from 13.75 to 57.42 J g^−1^ as the total PCM content increased from 20 to 50%.

**Table 4:** Thermal properties of composite PAN/PCM nanofibers between 5 and 45 °C.

Samples	Melting temp. (°C)	Latent heat of melting (ΔH_m_) (J g^−1^)	Onset of melting (°C)	Endset of melting (°C)	Crystallization temp. (°C)	Latent heat of crystallization (ΔH_c_) (J g^−1^)	Onset of crystallization (°C)	Endset of crystallization (°C)
Neat PAN	–	–	–	–	–	–	–	–
PAN-25PCM28	26.37	11.42	23.53	27.81	15.65/23.01	11.53	24.26	13.38
PAN-50PCM28	26.53	24.71	23.85	28.13	15.93/22.48	26.45	24.45	13.58
PAN-25PCM32	29.25	14.88	27.37	31.03	25.83	17.16	26.91	24.58
PAN-50PCM32	29.56	31.89	26.45	31.72	25.57	33.64	27.24	23.20
PAN-12.5PCM28-12.5PCM32	26.23/28.92	13.57	22.75	31.45	15.80/22.40/26.05	13.75	29.41	13.45
PAN-25PCM28-25PCM32	26.35/29.35	25.37	23.45	31.30	15.12/23.16/25.66	25.82	29.68	12.78
PAN-50PCM28-50PCM32	26.77/29.55	58.74	23.06	31.65	15.59/21.26/25.38	57.42	29.50	12.62

The enthalpy values of composite PAN/PCM nanofibers were lower than that of PCMs powder because PAN in the composite fibers did not have any contributions to enthalpy values at this temperature range. Additionally, the enthalpy values measured showed some variations from the theoretical enthalpy values. The theoretical enthalpy values calculated, and experimental enthalpy values obtained by DSC are presented in Table 5. Theoretically, the enthalpy values of the composite nanofiber mats were obtained by multiplying the latent heat of the PCM and the mass percentage of that PCM in the composite nanofiber mat^16^. From Table 5, it is clear that all the experimental values of enthalpy were lower than the corresponding theoretical values, and the efficiency of enthalpy (the ratio of the experimental value to the theoretical values) was less than 100%. The deviation of the experimental values from the theoretical values were attributed to the hindrance of thermal transitions of PCMs in the composite fibers by the presence of PAN acting as a diluent and also the quenching process during electrospinning^16–18,46,54–56^. During the electrospinning process, the evaporation of solvent occurs in milliseconds, and the molecules of PCMs may not have enough time to form well-defined structures in the composite fibers^17,20,22,23,54,57^. Besides, paraffins have a disadvantage that they have low thermal conductivity which usually falls short of providing the required heat exchange rate between the PCM and substrate^4^. The variations between the theoretical and actual values became less significant with the increase in the PCM content potentially due to the diluent effect of PAN surpassed by the larger amount of PCM.

**Table 5:** Theoretical and experimental enthalpy values.

	Theoretical value (ΔH_m_) (J g^−1^)	Experimental value (ΔH_m_) (J g^−1^)	%	Theoretical value (ΔH_c_) (J g^−1^)	Experimental value (ΔH_c_) (J g^−1^)	%
PAN-25PCM28	27.05	11.42	42.2	26.97	11.53	42.7
PAN-50PCM28	45.08	24.71	54.8	44.95	26.45	58.8
PAN-25PCM32	22.17	14.88	67.1	21.53	17.16	79.7
PAN-50PCM32	36.95	31.89	86.3	35.89	33.64	93.7
PAN-12.5PCM28-12.5PCM32	24.61	13.57	55.1	24.25	13.75	56.7
PAN-25PCM28-25PCM32	41.01	25.37	61.9	40.42	25.82	63.9
PAN-50PCM28-50PCM32	61.52	58.74	95.5	60.63	57.42	94.7

### Stability analysis—repeated heating and cooling cycles

**Figure 7:**
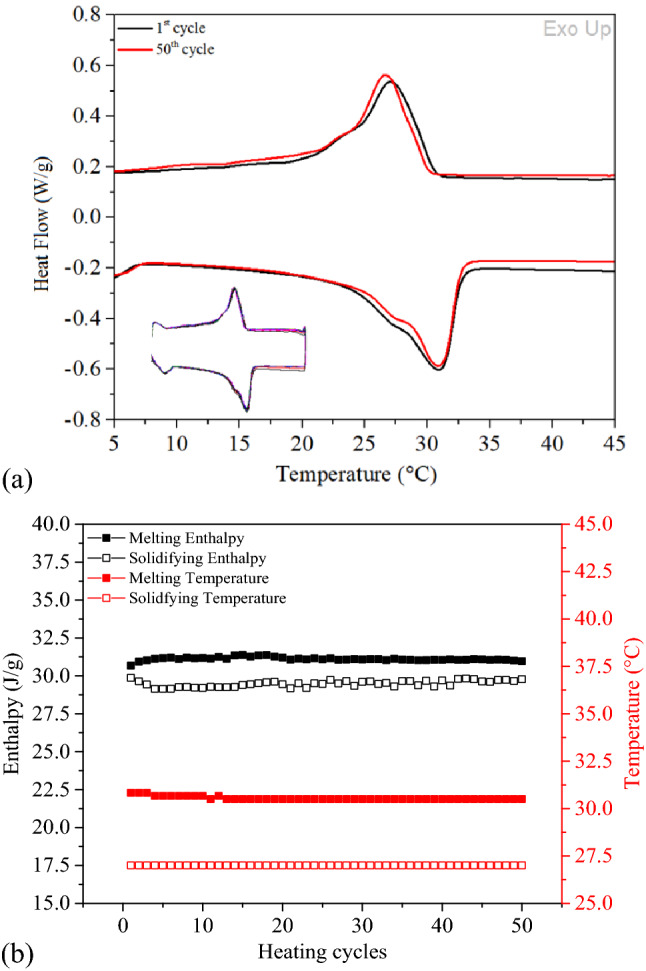
DSC curves of (**a**) 1st and 50th thermal cycles with the 50 consecutive thermal cycles in the inset (**b**) variations in storage and release enthalpies and thermal transition temperatures with consecutive thermal cycles for PAN-50PCM32 nanofiber mat.

Electrospinning enables it to produce novel thermoregulating materials which imparts form stability to PCMs in their molten form with the polymeric component of nanofibers acting as a scaffold for the PCMs. Composite PAN nanofiber mat containing 33% PCM32 (PAN-50PCM32) was tested regarding its thermal stability against 50 consecutive thermal cycles. The thermograms of 1st and 50th cycles are presented in Fig. 7a with the 50 cycles presented as an inset and the variations in storage and release enthalpies along with the variations in phase transition temperatures are presented in Fig. 7b. While the melting temperature decreased only about 0.3 ℃ at 50th heating cycle compared to 1st heating cycle, the solidifying temperature remained constant. No significant change was observed in the melting and solidifying enthalpies, which indicated the long-term stability of composite PAN nanofiber mats containing PCMs. The heating and cooling cycles applied repeatedly to composite PAN nanofibers containing PCMs clearly demonstrated that there was no thermal evaporation (thus no PCM loss) in the heating process and the thermal management functionality was stable for at least 50 cycles. Additionally, DSC thermal cycling test indicated that the composite nanofibers had good thermal reliability as the thermal properties of electrospun composite nanofibers were well retained after thermal cycling.

**Figure 8:**
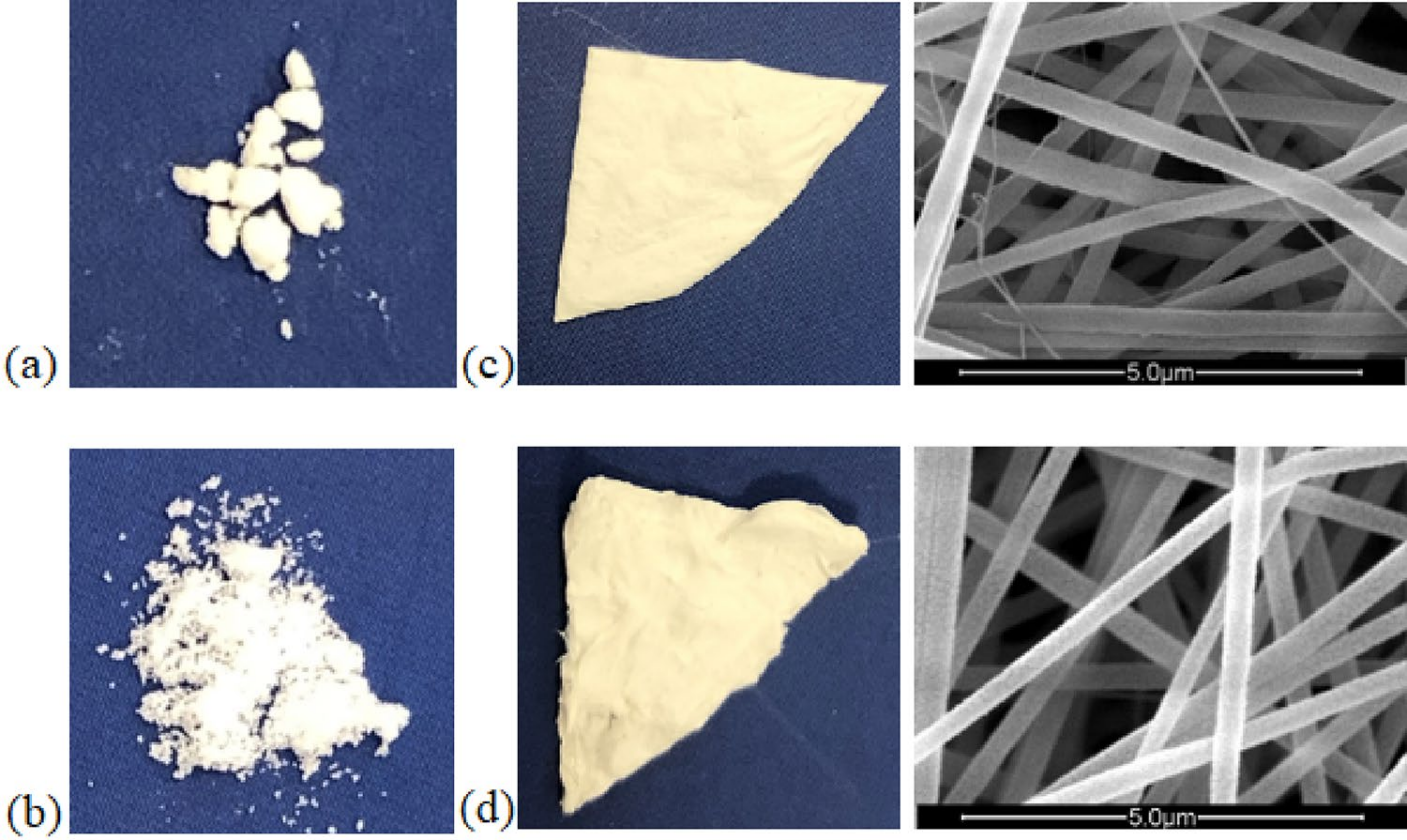
Photographs of (**a**) PCM28; (**b**) PCM32; photograph and SEM image of composite PAN-50PCM32 nanofiber mat (**c**) before (**d**) after 50 consecutive DSC thermal cycles.

The PCM particles were observed to be infused to each other (Fig. 8a,b) after DSC analysis. On the other hand, the composite nanofiber mat was observed to be stable with regards to its form after the consecutive thermal cycles. SEM was utilized to investigate the form stability of composite PAN nanofibers and SEM images obtained, illustrated in Fig. 8c,d, showed that the nanofibers maintained their cylindrical shapes after the 50 consecutive thermal cycles. The average nanofiber diameter was measured as 325.60 ± 38.30 and 315.07 ± 29.58 nm, before and after the thermal cycles, respectively. The composite fibers showed no obvious variations in the fibrous shape and the fiber diameter after the thermal treatment, which resulted from the protection and supporting effect of the polymer matrix in parallel with the work by Chen et al.^17^.

**Figure 9:**
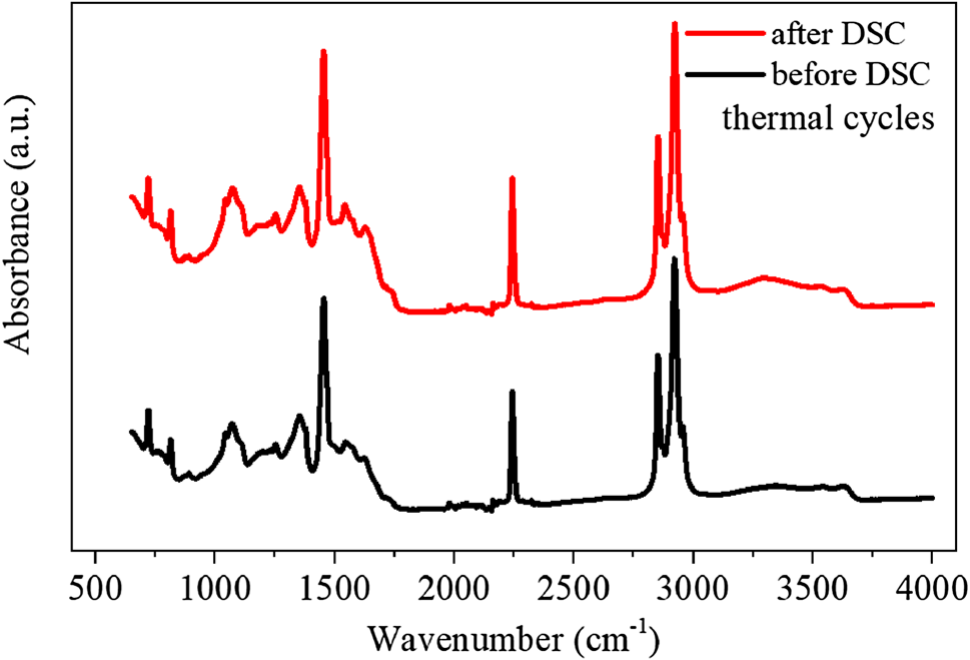
FTIR of composite PAN-50PCM32 nanofiber mat before and after 50 consecutive DSC thermal cycles.

FTIR was used to investigate the chemical structure of composite nanofiber mat before and after 50 consecutive DSC thermal cycles. The spectra (Fig. 9) demonstrated the structural stability of the PCM containing nanofibers by showing similar responses (with regards to the wavenumber and also peak intensity) particularly at ca. 3000 and 2800 cm^−1^, which were the vibrations of the C–H groups of the paraffinic PCMs.

### Thermal decomposition behaviour of composite nanofiber mats

**Figure 10:**
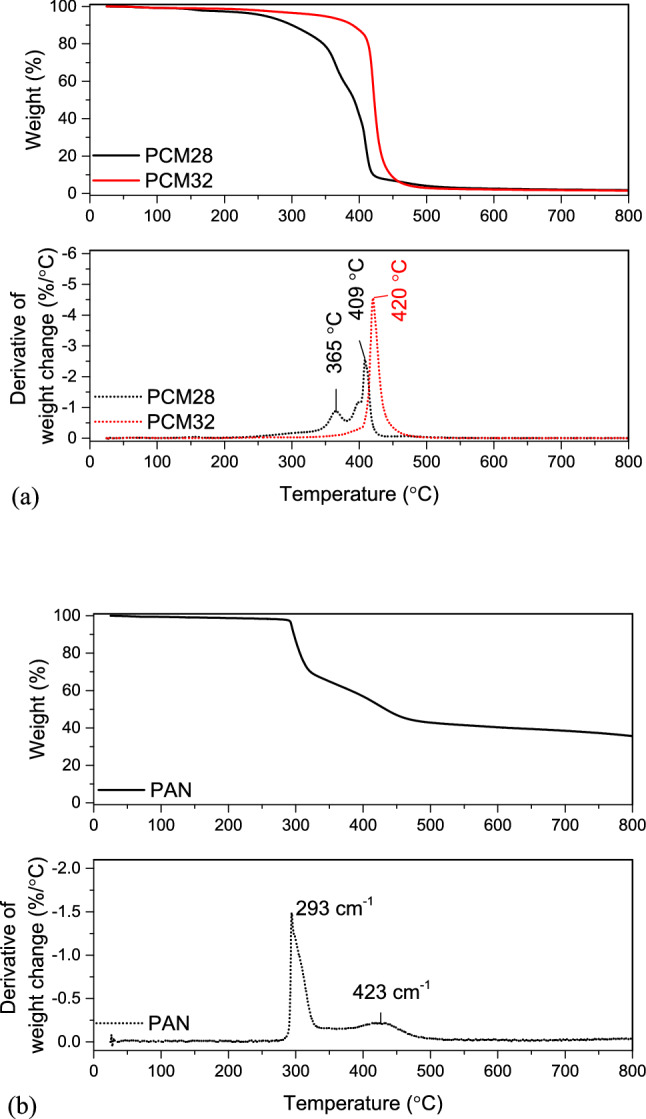
TGA and DTGA curves of (**a**) PCMs, (**b**) PAN.

**Figure 11:**
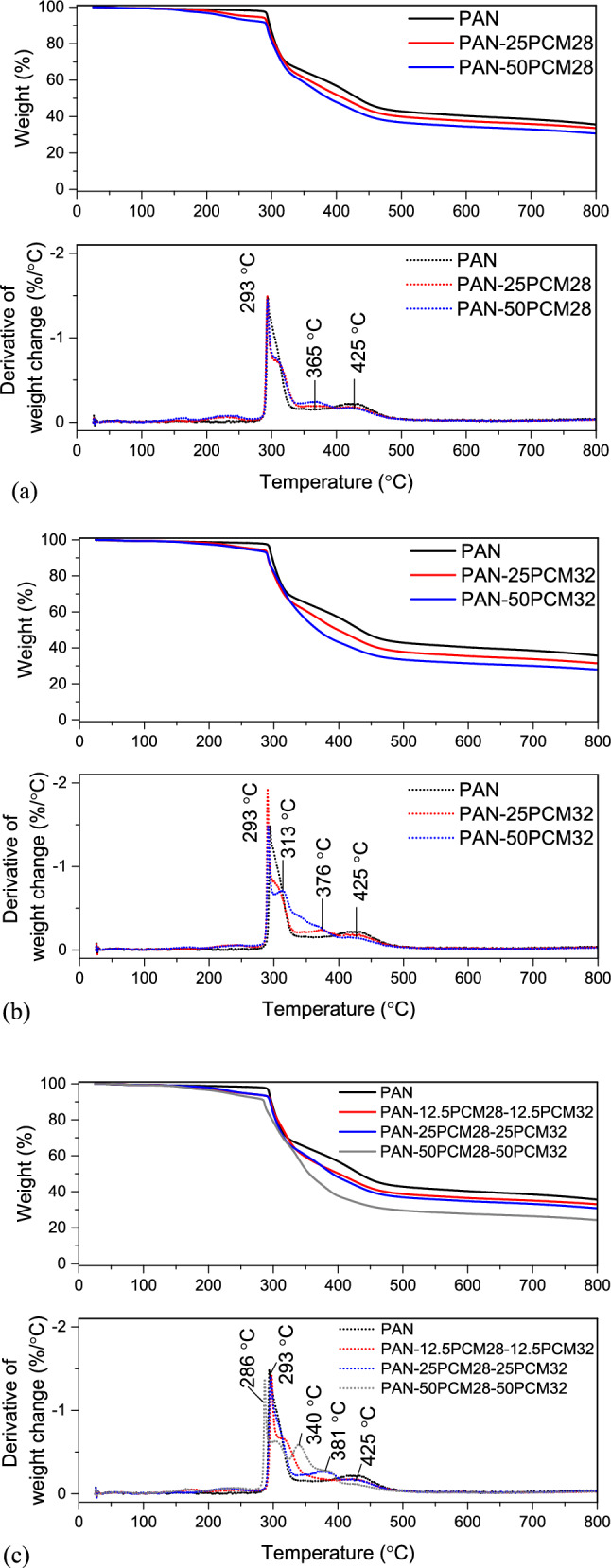
Thermal decomposition behaviours of composite PAN/PCM nanofiber mats in comparison to neat PAN nanofiber mat.

The thermal decomposition behaviour of PCMs, neat PAN and composite PAN/PCM nanofiber mats were evaluated using TGA in nitrogen atmosphere. While the TGA and DTGA curves of PCMs and neat PAN nanofiber mat are shown in Fig. 10, the TGA and DTGA curves of composite PAN/PCM nanofiber mats in comparison to neat PAN nanofiber mat are shown in Fig. 11. The weight loss values observed during different degradation regions (< 280 ℃, 280–332 ℃, 332–500 ℃, 500 ℃) along with the temperatures, at which the 5% weight loss took place (T_5%_) and the residue at 800 ℃ for PCMs, neat PAN and composite PAN/PCM nanofiber mats are presented in Table 6.

**Table 6:** TGA analysis of the PCMs, neat PAN and composite PAN/PCM nanofibers.

	T_5%_	Weight loss < 280 ℃	Weight loss 280–332 ℃	Weight loss 332–500 ℃	Weight loss > 500 ℃	Residue at 800 ℃
PCM28	258.83	7.09	9.03	79.83	2.29	1.77
PCM32	344.83	2.94	1.49	92.64	1.42	1.52
PAN	293.50	2.02	30.25	24.89	7.21	35.64
PAN-25PCM28	267.00	5.33	29.68	25.06	6.28	33.65
PAN-50PCM28	229.67	7.77	29.38	26.08	6.10	30.68
PAN-25PCM32	269.33	5.42	30.32	26.54	6.37	31.35
PAN-50PCM32	255.83	6.27	31.17	29.14	5.52	27.91
PAN-12.5PCM28-12.5PCM32	247.67	6.28	28.90	26.06	5.69	33.07
PAN-25PCM28-25PCM32	250.50	6.22	29.43	27.44	6.12	30.8
PAN-50PCM28-50PCM32	227.00	8.42	29.29	32.68	5.35	24.26

According to the TGA and DTGA results, PCM28 showed a two-step degradation with degradation peak (T_max_) values at 365.4 and 409 ℃ while PCM32 showed one-step degradation, with T_max_ at 420 ℃, the difference of which was likely due to the molecular weight differences of the PCM materials (Fig. 10a).

Figure 10b represents TGA analysis of neat PAN nanofibers. Neat PAN nanofibers showed a four-step weight loss profile from 25 to 800 ℃. In the first stage up to 280 ℃, there was a very slow and small weight loss. However, ~ 30% of weight loss from 280 to 332 ℃ was observed in the second stage, indicating that a significant chemical reaction took place, and volatile gases came out^58^. It is known that PAN polymer goes through intramolecular and intermolecular reactions mainly consisting of dehydrogenation, instantaneous cyclization, and crosslinking reactions, all of which are exothermic reactions. Among these three reactions, the predominant process is the cyclization of the nitrile groups into an extended conjugated ring system^59–64^. These cyclization reactions are shown up as a peak at 293 ℃ on the DTGA curve. During this step, the rate of weight loss was quite rapid. A further decrease of 24.89% weight loss was found from 332 to 500 ℃ in the third stage. In the last stage up to 800 ℃, a monotonous decrease of weight was observed with a total weight loss of 7.2%, leaving behind a residue of 35.64%.

The composite PAN/PCM nanofibers generally resembled the thermal behaviours of their components, namely the degradation of paraffins and the cyclization of PAN polymer chains. In the thermograms of composite PAN nanofibers with PCM28 (Fig. 11b), very small weight loss occurred up to 280 ℃. Between 280 and 332 ℃, weight loss due to the cyclization of PAN molecules was observed. Another very small peak between 332 and 396 ℃ with peak at around 365 ℃ appeared coming from the degradation of PCM28. The degradation peak observed at 409 ℃ for PCM28 (Fig. 10a) overlapped with the degradation peak of PAN in the temperature range between 396 and 500 ℃. The composite PAN nanofibers with PCM32, showed mainly similar thermal behaviour to that of composite nanofibers with PCM28. The maximum degradation temperature, which was observed at 420 ℃ on DTGA curve of PCM32 did not appear on DTGA of composite PAN/PCM32 nanofibers. Instead, a wider peak, merged with adjacent peaks, appeared at around 340 ℃, showing the earlier degradation of PCM32. This earlier degradation of PCMs were also observed on DTGA of composite nanofibers produced with simultaneous addition of both PCMs.

The temperature, at which the 5% weight loss took place (T_5%_) and the residue at 800 ℃ for PCMs, neat PAN and composite PAN/PCM nanofiber mats (Table 6) were also compared to evaluate their thermal degradation behaviours. The onset thermal degradation temperature of 5% weight loss for the composite nanofiber mats decreased from 293.50 ℃ for neat PAN fibers to about 227 ℃ when 50% PCMs were added to the nanofiber structure, which could be attributed to the early thermal decomposition of PCMs with lower thermal stability. The residues at 800 ℃ were 1.77 and 1.52% for PCM28 and PCM32, respectively. The residue at 800 ℃ decreased from 35.64% for neat PAN nanofibers to 24.26% with the addition of 30% PCMs to the nanofiber structure. As this residual was coming from the carbonization of PAN, its amount decreased as the PAN content decreased with the addition and increase in the amount of PCMs^20^.

### Hydrophobicity

Hydrophobicity of the composite nanofiber mats was evaluated by measuring water contact angle. In the contact angle measurement, a drop of a liquid is instilled onto the surface and then recorded by a camera. The video image is evaluated in order to determine the contact angle after a certain time. The larger the contact angle, the more hydrophobic the surface is. A surface is called hydrophobic if the water contact angle is larger than 90° and hydrophilic if the contact angle is smaller than 30°.

**Figure 12:**
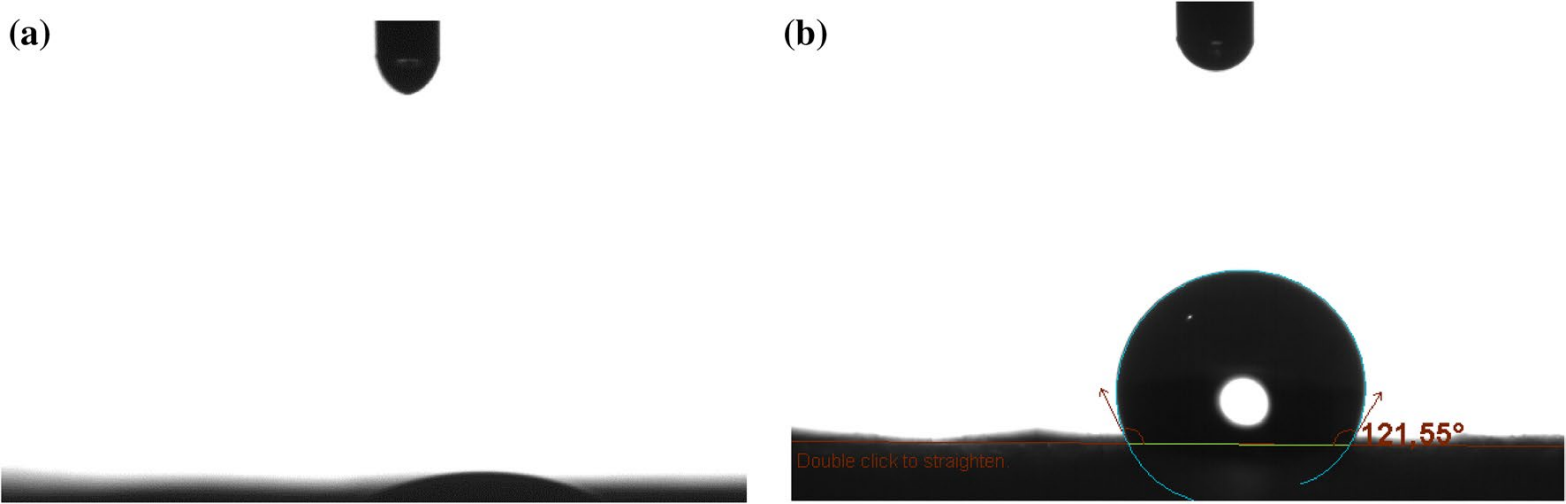
Contact angles obtained for (**a**) neat PAN and (**b**) composite PAN nanofibers with 50% PCMs (PAN-50PCM28-50PCM32).

The images of the drops taken by the camera are presented in Fig. 12. The composite nanofiber mats acquired hydrophobic properties with the addition of PCMs. While the water droplet was absorbed by neat PAN nanofiber mat in less than a second, the water contact angle was measured as 121.55° for the composite PAN nanofiber containing 50% PCMs. The hydrophobicity obtained by the addition of PCMs was due to the hydrophobic nature of PCMs, which were 100% pure paraffins and also due to the potentially increased roughness of the nanofiber surfaces in parallel with the study by Guo et al.^49^.

## Conclusions

This study was conducted with the aim of producing composite PAN/PCM nanofiber mats showing thermal management functionality. Uniform nanofibers were fabricated via electrospinning. They were white in colour. The composite nanofibers, which possessed smooth and cylindrical morphological structure with diameters of about 257–350 nm, showed different enthalpy values depending on the content and the type of PCMs. Increase was observed in average nanofiber diameter with addition of the additives. The enthalpy values of the composite fibers increased with the increase PCM content and the phase transition temperatures of the fibers had no obvious variations compared to that of PCMs. The latent heat and phase transition temperature of composite fibers were dependent on the type of the PCMs. A maximum of 50% PCM (based on total nanoweb weight) could be incorporated into the PAN nanofiber structure wherein the resulting composite nanofiber exhibited 58.74 J g^−1^ of enthalpy storage during heating with 95.50% efficiency compared to the theoretical value calculated based on the enthalpy values and contents of PCMs. The crystallization enthalpy was 57.42 J g^−1^ with an efficiency of 94.70%. It could be possible to obtain composite nanofibers showing thermoregulation ability over a wider temperature range by simultaneously adding PCMs with different melting points into PAN nanofiber structure. In addition, DSC thermal cycling test indicated that the composite nanofibers had good thermal reliability as the thermal properties of electrospun composite nanofibers were well retained after thermal cycling. The forms of the fibers were maintained after the cyclic thermal testing, which showed that nanofibers could be used as a shell enabling the formation of form-stable PCMs. The morphology, chemical structure, and composition of the composite fibers had no obvious variation after thermal treatments, which confirmed that the developed material showed form stable thermal management ability. The addition of PCMs also imparted hydrophobicity to the composite nanofiber mats. The composite nanofibers with thermal management functionality are suggested for use in active wear, undergarments, bedding, packaging materials, and insulation materials.
